# Bedaquiline reloading strategies following interruptions during daily dosing

**DOI:** 10.1128/aac.00063-26

**Published:** 2026-04-30

**Authors:** Nienke Kikstra, Stijn W. van Beek, Gary Maartens, James C. M. Brust, Elin M. Svensson, Simon E. Koele

**Affiliations:** 1 Department of Pharmacy, Pharmacology and Toxicology, Radboud Institute for Health Sciences, Radboud University Medical Center 6034, Nijmegen, the Netherlands; 2 Department of Rehabilitation, Radboud Institute for Health Sciences, Radboud University Medical Center 6034, Nijmegen, the Netherlands; 3 Pharmetheus AB 561081 , Uppsala, Sweden; 4 Wellcome Centre for Infectious Diseases Research in Africa, Institute of Infectious Disease and Molecular Medicine, University of Cape Town 656992, Cape Town, South Africa; 5 Division of Clinical Pharmacology, Department of Medicine, University of Cape Town, Cape Town, South Africa; 6 Divisions of General Internal Medicine and Infectious Diseases, Albert Einstein College of Medicine 2006, Bronx, New York, USA; 7 Department of Pharmacy, Uppsala University 8097, Uppsala, Sweden; Queen Mary University of London, London, United Kingdom

**Keywords:** pharmacokinetics, reloading, interruptions, bedaquiline, tuberculosis

## Abstract

Once-daily bedaquiline therapy is a WHO-listed alternative to thrice-weekly dosing for rifampicin-resistant tuberculosis, but, contrary to the thrice-weekly schedule, no guidance exists for treatment re-initiation after interruption. Using a population pharmacokinetic model, we simulated bedaquiline and M2 exposure under various interruption and reloading scenarios. Reloading strategies tailored to interruption duration restored bedaquiline exposure without clinically relevant increases in M2 peak concentrations, providing practical guidance for safe and effective treatment resumption of once-daily bedaquiline therapy.

## INTRODUCTION

Tuberculosis (TB), caused by *Mycobacterium tuberculosis*, is a major global health problem, causing an estimated 1.23 million deaths in 2024 (1). Control efforts are challenged by multidrug-resistant TB (MDR-TB).

Bedaquiline (BDQ) is a WHO-recommended core drug for MDR-TB treatment (2). It is characterized by extensive tissue distribution, >99.9% protein binding, and a terminal half-life of approximately 5.5 months (3). BDQ is metabolized to M2, which is associated with QT interval prolongation and the risk of Torsade de pointes (3–6).

The standard BDQ dosing regimen consists of 400 mg daily for 2 weeks, followed by 200 mg thrice weekly for 22 weeks (7, 8). The WHO also lists a once-daily alternative (200 mg daily for 8 weeks, followed by 100 mg daily for 18 weeks) (9–11). Moreover, a regimen with 400 mg daily for 2 weeks followed by 100 mg daily for 22 weeks is currently under investigation and aims to maximize early bactericidal activity (12).

Treatment interruptions occur in approximately one-third of patients receiving BDQ-containing regimens and are associated with poor outcomes and resistance development (13–16). Due to BDQ’s long half-life, safe and effective treatment resumption is complex. The required reloading period depends on residual exposure at restart. Insufficient reloading risks subtherapeutic concentrations and resistance, whereas excessive reloading may increase safety risks (17).

While reloading guidance exists for thrice-weekly dosing and is being prospectively evaluated, no optimized strategies are available for once-daily regimens (18–22). Therefore, this study aims to develop practical, safe, and efficacious BDQ reloading strategies for once-daily regimens using pharmacokinetic modeling.

Multiple interruption scenarios were evaluated. Pre-interruption treatment durations varied by 4-week increments: starting at 10–22 weeks for the 8-week loading regimen and at 4–16 weeks for the 2-week loading regimen. Interruption durations were 2, 4, 12, 24, and 52 weeks. Reloading strategies used the same doses used in the loading phase, with the 8-week loading regimen receiving 200 mg daily for 0, 1, 2, 4, 6, or 8 weeks, and the 2-week loading regimen receiving 400 mg daily for 0, 3, 7, or 14 days. A 0-duration strategy indicated a direct return to maintenance dosing.

A virtual cohort of 5,000 patients was generated. The covariate distributions of race (66% non-black, 34% black) and age (18–64 years, uniformly distributed) were based on clinical trials, while body weight and albumin were simulated using a population pharmacokinetic (PopPK) model (23–25).

Simulations used a previously published PopPK model (25). Full individual plasma concentration-time profiles of BDQ and M2 were generated for all patients across all interruption scenarios for both daily dosing regimens.

Efficacy and safety were assessed using weekly BDQ AUC (equation 1) and M2 *C*_max_ (equation 2), respectively, comparing values before interruption and after reloading. Weekly BDQ AUC was chosen as the driver of efficacy, and M2 *C*_max_ as the safety metric for its link to QT prolongation (25–28). Optimal reloading strategies were defined as those that minimize the absolute mean BDQ AUC deviation after reloading while ensuring that the mean M2 *C*_max_ after reloading did not exceed the mean M2 *C*_max_ before the interruption. Finally, these results were summarized to formulate practical optimized reloading strategies.


(1) $$BDQ\;AUC\;deviation\;after\;reloading=\;\frac{weekly\;BDQ\;AUC\;after\;reloading-weekly\;BDQ\;AUC\;before\;interruption}{weekly\;BDQ\;AUC\;before\;interruption\;}\;\times 100$$

(2) $$M2\;C_{max}\;deviation\;after\;treatment\;restart=\;\frac{M2\;C_{max}\;after\;reloading-M2\;C_{max}\;before\;interruption}{M2\;C_{max}\;before\;interruption\;}\;\times 100$$

Sensitivity analyses evaluated the robustness of the optimized strategies at the upper and lower boundaries of interruption-duration categories by comparing the longer and shorter reloading strategies. Details and results are described in the Supplemental material.

Data preparation and analysis were conducted in R v4.4.1, with simulations performed using NONMEM 7.5 with PsN and Pirana. R was used for post-processing and visualization.

**Fig 1:**
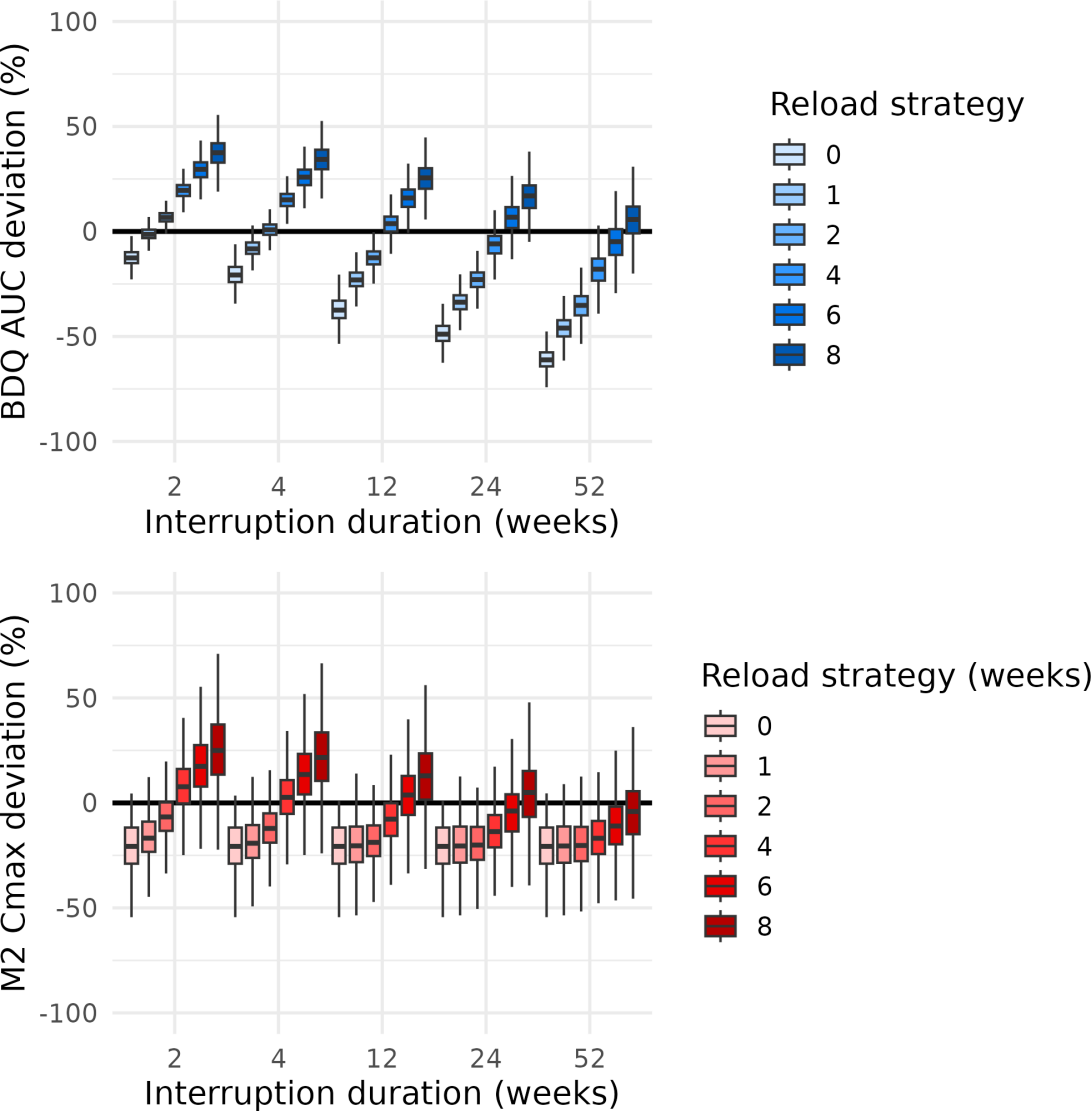
Boxplots of the BDQ AUC deviation after reloading (upper) and M2 *C*_max_ deviation after treatment restart (lower), stratified by interruption duration and colored by reloading strategy, for interruptions starting after 14 weeks of daily dosing BDQ treatment with an initial 8-week 200 mg once-daily loading phase. The boxplots depict the median and interquartile range, with the whiskers extending to the most extreme values within 1.5× interquartile range.

**Fig 2:**
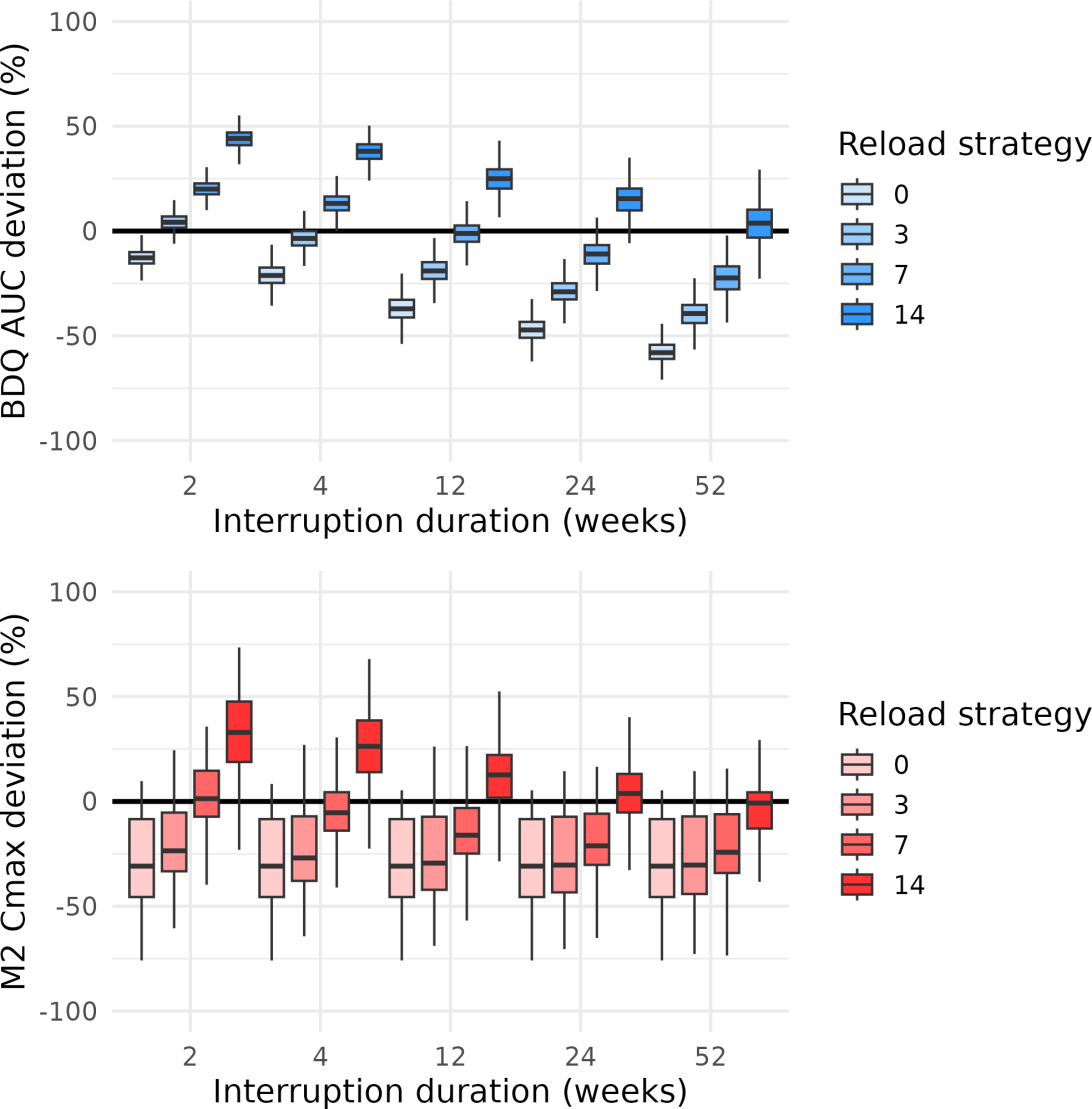
Boxplots of the BDQ AUC deviation after reloading (upper) and M2 *C*_max_ deviation after treatment restart (lower), stratified by interruption duration and colored by reloading strategy, for interruptions starting after 8 weeks of daily dosing BDQ treatment with an initial 2-week 400 mg once-daily loading phase. The boxplots depict the median and interquartile range, with the whiskers extending to the most extreme values within 1.5× interquartile range.

Across both daily dosing regimens, interruption duration was the primary driver of changes in BDQ exposure, while variations in treatment duration prior to interruption had minimal impact on BDQ AUC or M2 *C*_max_ deviations. Longer interruptions led to substantially lower BDQ AUC after reloading, whereas M2 *C*_max_ remained relatively stable. These trends were consistent across regimens, indicating that interruption length, rather than prior treatment duration, predominantly determines exposure reduction (Table S1). Therefore, a 6-week pre-interruption maintenance period was used to summarize the results (14 weeks total for the 8-week loading regimen; 8 weeks for the 2-week loading regimen). Figure 1 and 2 show weekly BDQ AUC and M2 *C*_max_ deviations after reloading for both daily dosing regimens.

These results were summarized with the interruption duration bands used for the optimized strategies being structured around the tested durations. Table 1 presents the optimized reloading strategies for both daily dosing regimens.

**TABLE 1:** Optimized bedaquiline reloading strategies following interruptions of once-daily 100 mg bedaquiline regimens

Interruption duration	Optimized reloading strategy
Daily dosing regimen starting with 8 weeks of loading	
<1 week	No reloading
1–3 weeks	1 week 200 mg bedaquiline daily
3–8 weeks	2 weeks 200 mg bedaquiline daily
8–18 weeks	4 weeks 200 mg bedaquiline daily
18–52 weeks	6 weeks 200 mg bedaquiline daily
≥52 weeks	8 weeks 200 mg bedaquiline daily
Daily dosing regimen starting with 2 weeks of loading	
<1 week	No reloading
1–4 weeks	3 days 400 mg bedaquiline daily
4–26 weeks	7 days 400 mg bedaquiline daily
≥26 weeks	14 days 400 mg bedaquiline daily

Sensitivity analyses confirmed the robustness of the optimized strategies. Across all tested interruption durations, BDQ exposure remained within an acceptable range, and M2 *C*_max_ deviations after reloading did not exceed pre-interruption concentrations to a clinically relevant extent (Table S2).

This study provides optimized reloading strategies for once-daily BDQ after treatment interruption. Using PK modeling, we simulated various interruption durations, onsets, and reloading approaches to assess BDQ and M2 exposure. Exposure after interruption was primarily driven by interruption duration rather than by prior treatment length, consistent with previous findings (20). Therefore, the optimized strategies are based solely on interruption duration.

The optimized reloading strategies were designed to restore BDQ exposure while minimizing M2-related safety risks, as confirmed by simulations; BDQ exposure returned to pre-interruption levels without clinically relevant increases in M2 *C*_max_. As M2 exposure remained similar or lower, no increased QTc risk is expected. Sensitivity analyses across the upper and lower interruption boundaries confirmed the robustness of these strategies, as exposure differences lower than 20% are generally not expected to have a clinically significant difference, and M2 *C*_max_ remained below pre-interruption levels (29).

Several limitations of this study should be considered. Interruption duration is often unknown at treatment resumption, complicating selection of the appropriate reloading strategy. Interruptions during the 8-week loading phase were not evaluated, although clinically plausible. Finally, the findings are based on PK simulations that simplify clinical reality and do not capture full individual variability, underscoring the need for clinical validation.

In conclusion, this study provides safe and effective optimized reloading strategies to support clinicians in restarting once-daily BDQ therapy following interruptions in tuberculosis treatment.

## SUPPLEMENTAL MATERIAL

Supplemental material aac.00063-26-s0001.docx Tables S1 and S2.

ASM does not own the copyrights to Supplemental Material that may be linked to, or accessed through, an article. The authors have granted ASM a non-exclusive, world-wide license to publish the Supplemental Material files. Please contact the corresponding author directly for reuse.
