# Supplementary material for: Bedaquiline reloading strategies following interruptions during daily dosing
Source: Antimicrob Agents Chemother. 2026 Apr 30;70(6):e00063-26. doi: 10.1128/aac.00063-26 (PMC13231907; doi:10.1128/aac.00063-26)
Supplement: Supplemental material — Tables S1 and S2. [file aac.00063-26-s0001.docx]

Supplementary material

Supplementary Table 1: The impact of BDQ treatment duration prior to interruption and the interruption duration on BDQ and M2 exposure with a respective reloading strategy of 0 weeks or days for the daily-dosing regimen starting with 8- and 2-weeks of loading.

| **Scenario type** | **BDQ treatment duration prior to interruption** | **Interruption duration** | **Weekly BDQ AUC deviation after reloading** | **M2 Cmax deviation after treatment restart** |
| --- | --- | --- | --- | --- |
| *Daily-dosing regimen starting with 8 weeks of loading* | | | | |
| Onset varying, duration fixed | 10 weeks | 4 weeks | -24.0% | -15.5% |
| Onset varying, duration fixed | 22 weeks | 4 weeks | -18.6% | -19.5% |
| Onset fixed, duration varying | 14 weeks | 2 weeks | -12.5% | -20.3% |
| Onset fixed, duration varying | 14 weeks | 52 weeks | -60.7% | -20.3% |
| *Daily-dosing regimen starting with 2 weeks of loading* | | | | |
| Onset varying, duration fixed | 4 weeks | 4 weeks | -24.9% | -25.1% |
| Onset varying, duration fixed | 16 weeks | 4 weeks | -19% | -26.2% |
| Onset fixed, duration varying | 8 weeks | 2 weeks | -12.6% | -29.0% |
| Onset fixed, duration varying | 8 weeks | 52 weeks | -57.5% | -29.0% |

Supplementary Table 2: Deviation in BDQ and M2 exposure following the optimized reloading strategies at the upper and lower limits of the interruption duration bands used in the optimized strategies for the once-daily dosing regimens starting with 8- and 2-weeks of loading with a respective 14- and 8-week treatment duration before interruption.

| **Interruption duration** | **Reloading strategy** | **Mean BDQ AUC deviation after reloading (%)** | **Mean M2 Cmax deviation after treatment restart (%)** |
| --- | --- | --- | --- |
| *Daily-dosing regimen starting with 8 weeks of loading* | | | |
| 1 week | 0 weeks | -7.2 | -20.3 |
| 1 week | 1 week | 3.2 | -12.4 |
| 3 weeks | 1 week | -4.8 | -17.7 |
| 3 weeks | 2 weeks | 3.6 | -10.0 |
| 8 weeks | 2 weeks | -7.1 | -16.5 |
| 8 weeks | 4 weeks | 8.3 | -4.4 |
| 18 weeks | 4 weeks | -2.1 | -12.0 |
| 18 weeks | 6 weeks | 10.4 | -1.4 |
| 38 weeks | 6 weeks | -0.5 | -8.9 |
| 38 weeks | 8 weeks | 9.8 | -1.1 |
| *Daily-dosing regimen starting with 2 weeks of loading* | | | |
| 1 week | 0 days | -7.0 | -28.9 |
| 1 week | 3 days | 9.5 | -15.6 |
| 4 weeks | 3 days | -3.4 | -24.7 |
| 4 weeks | 7 days | 13.0 | -5.3 |
| 26 weeks | 7 days | -12.7 | -20.0 |
| 26 weeks | 14 days | 13.3 | 1.3 |

**Approach sensitivity analysis**

A sensitivity analysis was performed to evaluate the robustness of the optimized reloading strategies for both loading regimens. The interruption durations selected for simulation correspond to the upper and lower boundaries of the interruption duration bands of the optimized strategies as presented in Table 1. Accordingly, for the daily-dosing regimen with an 8-week loading phase, interruption durations of 1, 3, 8, 18, and 38 weeks were simulated, while for the daily-dosing regimen with a 2-week loading phase, interruption durations of 1, 4, and 26 weeks were simulated. For boundary, the reloading strategies from the category directly below and above were simulated to assess their appropriateness. In the daily-dosing regimen with 8 weeks of loading, this included 0 (no reloading), 1, 2, 4, 6, and 8 weeks of reloading, whereas in the daily-dosing regimen with 2 weeks of loading, 0 (no reloading), 3, 7, and 14 days were tested. For example, for interruptions shorter than 1 week, both 0 and 1 week of reloading (daily-dosing regimen with an 8-week loading phase) or 0 and 3 days of reloading (daily-dosing regimen with a 2-week loading phase) were compared to determine the most suitable reloading strategy. Interruptions were simulated to start after 14 weeks of BDQ for the daily-dosing regimen with 8 weeks of loading, and after 8 weeks of treatment in the regimen starting with 2 weeks of loading.

**Results sensitivity analysis**

The deviation in BDQ and M2 exposure following the optimized reloading strategies at the upper and lower limits of the interruption duration bands used in the optimized strategies was evaluated to check the robustness of the optimized strategies. Across all tested interruption durations for both once-daily BDQ regimens, BDQ exposure remained within an acceptable range and mean M2 Cmax deviations after treatment restart did not exceed the mean M2 Cmax prior to the interruption to a clinically relevant extent (Supplementary Table 2).
